# A spatial analysis of the associations between housing eviction and alcohol-related hospitalizations in Pennsylvania

**DOI:** 10.1016/j.healthplace.2026.103632

**Published:** 2026-02-16

**Authors:** Michelle Dougherty, Robert W.S. Coulter, Sarah L. Pedersen, Maya I. Ragavan, Sara E. Baumann, Christina Mair

**Affiliations:** a Department of Behavioral and Community Health Sciences, School of Public Health, University of Pittsburgh, 130 DeSoto Street, Pittsburgh, PA, 15261, USA; b Center for Social Dynamics and Community Health, School of Public Health, University of Pittsburgh, 130 DeSoto Street, Pittsburgh, PA, 15261, USA; c Department of Psychiatry, University of Pittsburgh, 3811 O'Hara St, Pittsburgh, PA, 15213, USA; d Division of General Academic Pediatrics, University of Pittsburgh and UPMC Children's Hospital of Pittsburgh, 3414 Fifth Avenue, Pittsburgh, PA, 15213, USA

**Keywords:** Alcohol, Housing eviction, Spatiotemporal analysis

## Abstract

**Purpose:**

Alcohol consumption has substantial negative health consequences and those who experience homelessness bear a disproportionate burden of these consequences. Housing eviction is a widespread, modifiable cause of homelessness and housing insecurity more broadly. Understanding the associations between eviction and alcohol-related harms could reveal important prevention opportunities.

**Methods:**

We examined spatial patterns of inpatient hospitalizations for alcohol use disorder (AUD) and harms that are partially attributable to alcohol assumption, including assault, intimate partner violence (IPV), and self-harm at the ZIP code level in Pennsylvania. Using Bayesian hierarchical space-time misalignment models, we examined the associations between ZIP code-level eviction filing rates and hospitalizations for these conditions, reporting relative rates (RR) and 95% credible intervals (CIs). We stratified our analyses across two time periods, 2018–2019 (n = 2901 ZIP code-years) and 2020–2022 (n = 4356 ZIP code-years).

**Results:**

Eviction filings were associated with increased hospitalizations for AUD; a one percentage point increase in the eviction filing rate was associated with a 1.4% (95% CI: 1.011 – 1.018) and 1.1% (95% CI:1.007–1.014) increase in AUD hospitalizations in 2018–2019 and 2020–2022, respectively. The associations between eviction filings and hospitalizations for IPV (RR: 1.018, 95% CI: 1.005–1.031) and hospitalizations for assault (RR: 1.033, 95% CI: 1.002, 1.065) were only well-supported during 2020–2022.

**Conclusions:**

This study highlights the potential for leveraging housing eviction interventions for the prevention of alcohol-related harms, especially AUD. Future research should examine the mechanisms linking eviction, alcohol consumption, and related harms, and measure the alcohol-related impacts of eviction prevention interventions.

## 1. Introduction

One of the most commonly consumed substances in the world, alcohol imposes a significant public health burden, leading to 4.7% of deaths worldwide in 2019 (World Health Organization, 2024). In the U. S. specifically, there were approximately 140,000 alcohol-related deaths annually from 2015 to 2019 (Center for Disease Control and Prevention, 2022). Individuals experiencing homelessness are disproportionately impacted by alcohol, with rates of alcohol use disorder (AUD) and alcohol-attributable mortality at least four and six times that of the general population, respectively (Baggett et al., 2015; North et al., 2010; Substance Abuse and Mental Health Services Administration, 2011; Tsai, 2018; Tsai et al., 2014). Housing programs in the U.S. often prioritize those experiencing chronic homelessness, as opposed to housing insecurity more broadly, defined as lack of stable occupancy in a decent, safe, and affordable living space (U.S. Department of Housing and Urban Development, 2025a; Watson and Carter, 2020). Efforts to address substance use disorder, including AUD, in combination with housing insecurity have also focused those experiencing chronic homelessness through housing first approaches (i.e., low-barrier housing without the requirement for abstinence) (Collins et al., 2012; Woodhall-Melnik and Dunn, 2016). While important, those experiencing chronic homelessness constitute a small portion of the larger population experiencing housing insecurity. As of 2023, 653,104 individuals experienced homelessness and 143,105 experienced chronic homelessness, while 21.6 million rental households were cost burdened (i.e., paid more than 30% of household income on rent) and 44% of renter households felt pressured to move in the past 6 months (e.g., due to rent unaffordability, eviction threat, poor housing or neighborhood conditions) (Joint Center for Housing Studies, 2023; Reynolds and Burton, 2023; U.S. Department of Housing and Urban Development, 2023). Better understanding how alcohol consumption and related harms may be connected to less severe forms of housing insecurity may unveil opportunities for preventing these harms among those at risk.

Housing eviction – instances in which landlords force renters to move – is a modifiable cause of housing insecurity. (Collinson and Reed, 2018; Desmond, 2012). Renters experiencing housing eviction and the threat of housing eviction (i.e., anticipating an eviction notice or forced move) are exposed to different dimensions of housing insecurity linked to poor health outcomes, including forced mobility, decreased accessibility, as well as poorer housing quality and neighborhood conditions (e.g., greater poverty, more crime) (Desmond et al., 2015; Swope and Hernández, 2019). Housing eviction may increase alcohol consumption and related harms through these dimensions. Unaffordable housing costs, loss of housing, neighborhood crime and poverty, and housing dilapidation can cause intense mental distress, which is associated with increased binge drinking and AUD (Bernstein et al., 2007; Cerdá et al., 2010; Hill and Angel, 2005; Keyes et al., 2012; Mulia et al., 2014; Murphy et al., 2014). Beyond the individual level, displacement and community uprooting due to eviction can decrease social cohesion and social capital, creating conditions (e.g. less social accountability and bystander intervention) where violence is more likely to occur (Schwartz et al., 2024). A high neighborhood-level eviction rate could disrupt supportive social networks that protect against negative drinking consequences (Bryden et al., 2013; Murphy et al., 2014; Schwartz et al., 2024; Tam et al., 2021). It may also increase the overall salience of eviction threat and the perception that one's neighborhood is unfairly targeted (e.g., due to racial stigma), increasing both general psychosocial and identity-based stressors, which may increase alcohol consumption and risk of AUD (Arcaya et al., 2014; Keyes et al., 2012a; Schwartz et al., 2024). Eviction moratoria, rental assistance, landlord-tenant mediation, and access to civil legal aid have all shown success in reducing eviction (Ellen et al., 2021; Kong et al., 2022; Marçal et al., 2023). Understanding which alcohol-related outcomes are related to eviction could elucidate whether and how we can leverage these interventions to reduce alcohol-related harms.

Few studies have examined the associations between eviction and harms fully attributable to alcohol, especially AUD. Bradford and Bradford (2020) found that eviction rates were positively associated with alcohol-related mortality at the county level in urban contexts, but this study was limited to alcohol poisoning deaths. More studies have examined whether eviction may be associated with an increase in harms that are partially attributable to alcohol, such as those related to interpersonal violence, although substantial knowledge gaps remain. There is evidence that eviction is associated with increased suicide ideation and mortality (Elbogen et al., 2021; Guzman-Parra et al., 2023; Peek-Asa et al., 2021; Rojas and Stenberg, 2016) and intimate partner violence (IPV) (Benson-Goldsmith et al., 2024; Goldenberg et al., 2023; Groves et al., 2022; R Wilson et al., 2021; Schwab-Reese et al., 2016); however, all of these studies were conducted at the individual level, a key gap given that eviction, suicide, and IPV risk mechanisms also operate at an ecological level (e.g., neighborhood level) (Assari, 2013; Cramer and Kapusta, 2017; Schwartz et al., 2024; Wray et al., 2011). By contrast, several studies have focused on eviction and violent crime have considered both the ecological- and individual-level mechanisms (Desmond and Gershenson, 2017; Gomory and Desmond, 2023; Kirk, 2021; Semenza et al., 2021, 2022). However, all these studies were focused on specific urban areas, an important gap as there have been greater increases in eviction outside urban areas over time and the relationship between eviction and alcohol-related outcomes may vary across urban vs. rural areas (Bradford and Bradford, 2020; Rutan et al., 2023).

The COVID-19 pandemic significantly impacted both eviction and alcohol-related outcomes. The medium-to long-term impacts of the pandemic are still emerging; however, both rates of heavy drinking and alcohol-related hospitalizations increased during the early phase of the pandemic and remained elevated through at least 2022 (Ayyala-Somayajula et al., 2024; Schmidt et al., 2021; Yaseen et al., 2025). Interpersonal violence also increased during this time period (O'Neill et al., 2023; Ssentongo et al., 2023). In particular, physical, sexual, and psychological IPV victimization and perpetration increased, with several studies linking these increases to social isolation, unemployment, and financial strain (Bhuptani et al., 2022; McNeil et al., 2023). Although there was a federal moratorium on eviction in the U.S. from March 27, 2020 through August 26, 2021 (Centers for Disease Control and Prevention, 2021; Congressional Research Service, 2021), eviction filings persisted and remained a stressor. During 2020, an increasing number of tenants delayed their rental payments and those who did so had seven-fold increased odds of perceived eviction risk and three times the odds of past two-week suicide ideation (Tsai et al., 2022). Altogether, it is possible that any relationship between eviction filings and alcohol-related outcomes may have changed during the pandemic in light of increased financial strain, psychological distress, and social isolation.

In summary, there is a paucity of research on housing eviction and alcohol-related harms, especially AUD. Better understanding these relationships may unveil opportunities for preventing these harms among those at risk for homelessness, which is associated with large increases in the risk of alcohol-related morbidity and mortality. The primary aim of this study is to examine the spatial patterning of inpatient hospitalizations for AUD, assault, IPV and self-harm and the associations between these spatial patterns and ZIP code-level eviction filing rates across Pennsylvania (PA). A secondary aim is to assess whether these associations vary before vs. during the COVID-19 pandemic.

## 2. Methods

We used exploratory spatial data analysis methods to visualize spatial clustering of inpatient hospitalizations for AUD, assault, IPV and self-harm at the ZIP code level across PA by year. Next, we used Bayesian space-time misalignment models to examine the associations between ZIP-code level eviction filing rates and counts of inpatient hospitalizations for these conditions, adjusting for community sociodemographic characteristics. To examine these associations absent pandemic-related influences and to explicitly compare how associations may differ pre- vs. post-pandemic, we stratified our analyses, running separate models for 2018–2019 vs. 2020–2022 and qualitatively compared associations across these periods by assessing whether point estimates and distributions changed in a meaningful way (e.g., if 95% credible intervals shift to no longer include 1). The institutional review board at [redacted] reviewed this study and determined that it was exempt.

### 2.1. Data sources and measures

We linked all data for this study at the ZIP code level by year. ZIP codes correspond to post office delivery areas and can include point-based areas, such as post office boxes. We spatially joined ZIP codes to create contiguous areas (e.g., by merging points such as post office boxes into adjacent areas), resulting in year-specific shapefiles with 2901 space-time units in 2018–2019 and 4356 space-time units in 2020–2022.

We obtained inpatient hospital discharge data for all hospitals and freestanding ambulatory centers in PA (excluding Veteran's Affairs and other federally-run hospitals) (Pennsylvania Health Care Cost Containment Council, 2024). These data included information on each patienťs ZIP code, principal diagnosis codes, and up to 17 secondary diagnosis codes. We used ICD-10 codes to identify hospitalizations for AUD, assault, IPV, and self-harm, creating aggregate counts of each of these hospitalization types by ZIP code (See Supplement for ICD-10 codes). Hospitalizations for these conditions were counted if the associated ICD-10 code was included either as a primary or secondary diagnosis code. For assault, IPV, and self-harm, hospitalizations were included regardless of whether fully-alcohol attributable conditions were also diagnosed using a primary or secondary code as prior literature shows that a substantial portion of morbidity and mortality for these conditions is attributable to alcohol consumption (Alpert et al., 2022; Caetano et al., 2001; Center for Disease Control and Prevention, 2022; Kuhns et al., 2013). Of 7,829,185 inpatient hospitalizations in PA in 2018–2022, we excluded 5.0% because the residential ZIP code was invalid or the patient was living outside of PA.

We obtained eviction filing court records from the Magisterial District Judge (MDJ) System from 2018 to 2022 for all of PA, with the exception of Philadelphia, which we obtained from Philadelphia Legal Assistance (Philadelphia Legal Assistance, 2024; The Unified Judicial System of Pennsylvania, 2025).^1^ We reviewed eviction records and excluded cases in which the ZIP code of the defendant (i.e., tenant) was missing, invalid or outside of PA, and limited cases to residential eviction by searching defendant names for key terms associated with businesses (e.g., corporation, LLC) in line with best practices (Desmond et al., 2018). Of 460,669 cases, 99.2% (n = 456,948) were included for analysis across 2018–2022. We included eviction records regardless of what the case disposition (i.e., court ruling) was as these fields were often incomplete. Eviction filings are often used as proxy for forced moves due to eviction and are indicative of eviction threat, an important health exposure onto itself (Garboden and Rosen, 2019; Gromis et al., 2022). We calculated eviction filing rates by aggregating counts of eviction filings per ZIP code per year and dividing this by the number of rental households in that ZIP code, obtained from the U.S. census, described next.

For socioeconomic and demographic characteristics, we used 5-Year American Community Survey (ACS) tract-level estimates for each year of analysis and extrapolated estimates to the ZIP code level. To do this, we assigned census tract-level estimates to the census blocks within each track, then spatially linked ZIP codes to census blocks to calculate population weights (i.e., the proportion of each block's population that was within each ZIP code), which we then applied to the tract-level estimates associated with each block, summing these within ZIP codes (i.e., similar to a weighted average).

We included socioeconomic and demographic variables hypothesized to confound the relationship between ZIP code-level eviction filing rates and our outcomes of interest based on prior literature (Desmond and Gershenson, 2017; Mair et al., 2013, 2020; Skinner et al., 2024; Sumetsky et al., 2020). These included the percent of total population across age groups (percent <18, 18–24, 25–44, 45–64, and ≥65), percent male, race and ethnicity distributions (percent Non-Hispanic or Latino White, Non-Hispanic or Latino Black, Hispanic or Latino, and all other races and ethnicities), percent of population age ≥15 married, percent of total workforce unemployed, percent of total population with incomes below the federal poverty level, median household income per $10,000, percent of those age ≥25 with a college degree, and the percent insured. We categorized population density into equal quintiles across PA (corresponding to 0–56.5, 56.6–141.6, 141.7–414.4, 414.5–1754.9 and >1754.9 people per mile^2^ for 2018–2019 and 0–55.9, 60.0–140.1, 140.2–407.0, 407.1–1742.0, and >1742.0 people per mile ^2^for 2020–2022) and divided these values by 100 to ensure that credible intervals would be wide enough to interpret. We also used indicators of the local housing environment from the ACS, including the percent of households experiencing rent burden (paying >30% of income on rent) and the percentage of vacant housing units.

To capture healthcare utilization and availability, we calculated the number of hospitalizations per 100 people in each ZIP code per year. We also obtained the locations of all hospitals and facilities providing substance use and/or mental health (SUMH) treatment in PA (Pennsylvania Spatial Data Access, 2025; Substance Abuse and Mental Health Services Administration, 2025). Using QGIS (version 3.34.11), we aggregated the number of hospitals and the number of facilities providing SUMH treatment by ZIP code by year (Open Source Geospatial Foundation Project, 2025). Since these health care resources were sparsely distributed (only 13% of ZIP codes had a hospital and 25% had an SUMH treatment facility) we created binary indicators for these variables to capture if there were one or more hospital or SUMH treatment facility per ZIP code.^2^

We obtained a list of all active liquor licenses from the Pennsylvania Liquor Control Board (PLCB) (PLCB+ Online Regulatory System, 2025). We restricted this dataset to establishments regularly selling alcohol for on- or off-premise consumption, including restaurants, beer distributors, breweries/brewery pubs, limited wineries and distilleries, taverns, and eating places, based on the descriptions of these establishment types provided by the PLCB (Pennsylvania Liquour Control Enforcement, 2025). Since this dataset contained multiple rows per license number documenting the history of each license, we cleaned these data to create a listing of unique alcohol outlet addresses. The locations of state-run liquor stores were obtained separately from the PLCB annual reports (Pennsylania Liquour Control Board, 2019). We geocoded addresses of alcohol outlets using Geocodio (2025). Of 13,280 addresses, 3.9% could not be geocoded or were outside of PA, leaving the addresses of 12,765 outlets for analysis. We identified outlets selling alcohol for primarily off-premise consumption, including state-run liquor stores, beer distributors and eating places, in line with prior analyses of alcohol outlets in PA (Grubesic et al., 2013). Using QGIS (version 3.34.11), we generated an aggregate count of all outlets and divided this by the area (in miles^2^) of each ZIP code to calculate overall outlet density. We also calculated the proportion of all alcohol outlets per ZIP code selling alcohol for off-premise consumption.

Eleven ZIP codes had populations less than five. For these ZIP codes, we changed the ZIP code population count to five and replaced the values of other variables with the year-specific statewide mean to ensure there was a non-zero risk of hospitalizations in all ZIP codes. Finally, we included year-specific dummy variables in our analyses to control for differences across years not explained by other covariates and likely due to temporal trends.

### 2.2. Analysis

We explored spatial patterns of hospitalizations by year for AUD, assault, IPV, and self-harm using GeoDA (Anselin et al., 2006). For each of these variables, we calculated the Global Moran's I to assess spatial autocorrelation. To visualize local clusters and hotspots we used G*, a local indicator of spatial association that compares the average value of each ZIP code to the global average. We used a queen contiguity matrix for all these analyses.

Using Bayesian hierarchical space-time misalignment models, we examined the relationship between eviction filings and each outcome of interest by ZIP code-year. Since ZIP code boundaries shift by year, we accounted for spatial misalignment between years by including separate conditional autoregressive (CAR) random spatial and nonspatial effects for each space-time unit in our models (Zhu et al., 2013). We assigned uninformed priors for all fixed and random effects. We used Poisson regression for all models as all outcomes were counts.


$$Y_{i,t}|\mu_{i,t}∼\mathrm{Poisson}\left(E_{i,t}exp\left(\mu_{i,t}\right)\right)$$

In this equation, $Y_{i,t}$ is the count of hospitalizations for each ZIP code i in year t. $E_{i,t}$ is the expected count of hospitalizations under the assumption that these hospitalizations are distributed directly proportional to total ZIP code population. The relative rate for each space-time unit is represented by $exp\left(\mu_{i,t}\right)$. The log relative rate $\mu_{i,t}$ is modeled as:

$$\mu_{i,t}=\beta X_{i,t}+\phi_{i,t}+\theta_{i,t}$$

In this equation, β is the vector of coefficient estimates for each fixed effect, while $X_{i,t}$ is the matrix of fixed effects. $\phi_{i,t}$ is the vector of CAR spatial random effects, and $\theta_{i,t}$ is the vector of nonspatial random effects.

We used the R-INLA package to fit the models using integrated nested Laplace approximation (INLA), a deterministic approach used as an accurate alterative of Markov Chain Monte Carlo (MCMC) approaches (Carroll et al., 2015; Held et al., 2010; Lindgren and Rue, 2015). For each outcome, we fit two models, one using 2018–2019 data and one using 2020–2022 data. In building models, we used a stepwise approach, assessing each fixed effect as it was added and removing fixed effects that were not well-supported, assessing whether removal affected other fixed effects. Since these models adjust for changes in ZIP code boundaries across years, they do not allow for a longitudinal assessment of changes within ZIP codes over time. All Bayesian space-time models were run in R version 4.4.2 (R Core Team, 2024).

## 3. Results

From 2018 to 2022 there were 7,438,646 inpatient hospitalizations in PA. Of these, 5.82% (n = 358,367) were for AUD, 0.05% (n = 3394) were for assault, 0.15% (n = 11,223) were for IPV, and 0.05% were for self-harm (n = 3685) (based on either primary or secondary diagnosis codes). As shown in Table 1, there was considerable heterogeneity in hospitalization counts across ZIP codes, especially with respect to AUD hospitalizations (mean: 52, range: 0–888 in 2018–2019), and IPV hospitalizations (mean: 1.57, range: 0–111 in 2018–2019). The average eviction filing rate was 4.70% of rental households in 2018–2019 and 3.33% in 2020–2022 and this also varied across ZIP codes considerably (e.g., SD: 5.24%, range: 0% - 65.91% in 2018–2019). The other variables included in analysis were similarly heterogenous across ZIP codes.

Exploratory spatial data analysis showed significant spatial autocorrelation for all four outcomes in all years: AUD hospitalizations (Moran's I range: 0.140–0.234, all p values < 0.001), assault hospitalizations (Moran's I range: 0.113 – 0.305, all p values < 0.006), IPV hospitalizations (Moran's I range: 0.106 – 0.302, all p values < 0.001), and self-harm hospitalizations (Moran's I range: 0.062–0.278, all p values < 0.008).

**Fig. 1.**
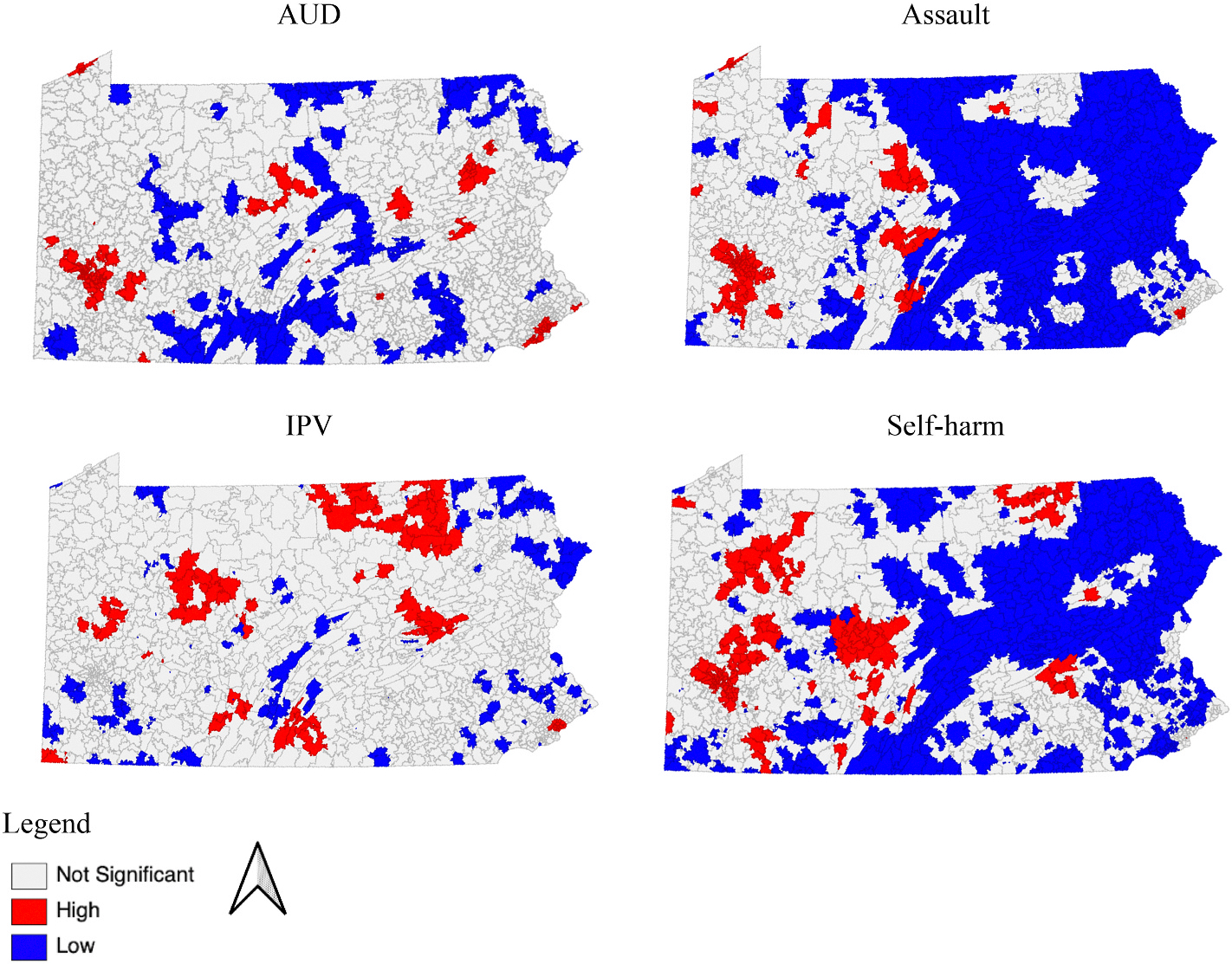
Local Indicator of Spatial Association G* of Alcohol-related Hospitalizations by ZIP code in Pennsylvania in 2018.

Local indicator of spatial association G* maps reveal distinct patterns of spatial clustering for each outcome, as shown in Fig. 1, which shows G* maps for 2018 only as the overall pattern of cold and hot spots remains consistent across years for these outcomes. AUD hospitalization hotspots largely corresponded to urban areas in Pennsylvania (Philadelphia, Pittsburgh, Erie, and Scranton/Wilke-Barre), although there were also hotspots in more rural central regions of the state. Similarly, there were assault hospitalization hotspots in Philadelphia, in and around Pittsburgh, Erie, and in the rural central region of the state with notable cold spots in the eastern half of the state. Although there was a hotspot for IPV hospitalizations in Philadelphia in all years, most of the other hotspots for IPV hospitalizations were in rural central and northeastern portions of the state. Self-harm hospitalization hotspots were largely in the western half of the state and rural areas to the north and east of this region.

The results of Bayesian space-time misalignment models for alcohol-related inpatient hospitalizations in Pennsylvania for 2018–2019 vs. 2020–2022 are shown in Tables 2 and 3. Overall, the association between eviction filings and AUD hospitalizations was consistently well supported across both time periods. A one percentage point increase in the eviction filing rate was associated with a 1.4% (95% CI: 1.011 – 1.018) increase in AUD hospitalizations in 2018–2019 and a 1.1% (95% CI:1.007–1.014) increase in AUD hospitalizations 2020–2022. The association between eviction filing rates and self-harm hospitalizations was consistently not well supported across both time periods. The effect of eviction filings for both assault hospitalizations (RR:1.033, 95% CI: 1.002, 1.065) and IPV hospitalizations (RR: 1.015, 95% CI: 1.002–1.028) was well supported in 2020–2022, but not 2018–2019.

For AUD hospitalizations, a higher proportion of those age 45–64, lower median household income, increased alcohol outlet density, a higher proportion of off-premise outlets, increased percent insured, a lower proportion of married residents, and whether a ZIP code had an SUMH facility were associated with increased hospitalizations across time periods. The effect of the percent unemployment varied notably across time periods and was associated with increased hospitalizations for AUD in 2020–2022 and decreased hospitalizations 2018–2019. The association between the percent of residents with incomes below the poverty level and AUD hospitalizations was well-supported in 2020–2022, but not in 2018–2019.

The percentage of residents who were Hispanic or Latino and the percent married were associated with decreased hospitalizations for assault across both time periods. Alcohol outlet density had a well-supported positive association with assault hospitalizations, but only during 2018–2019. Similar to assault, the percentage of residents who were Hispanic or Latino and the percentage married were associated with decreased hospitalizations for IPV, while alcohol outlet density was associated with a well-supported increase in IPV hospitalizations, but only in 2020–2022. The relationship between economic variables (unemployment, poverty, and median household income) and IPV hospitalizations were well supported during 2020–2022, but not 2018–2019; in 2020–2022. The percentage of Hispanic or Latino residents had a well-supported negative association with self-harm hospitalizations across both time periods, similar to other outcomes. Having an SUMH facility in a ZIP code was associated with increased self-harm hospitalizations, but this relationship was only well-supported in 2018–2019. The overall number of hospitalizations per 100 people was associated with increased hospitalizations for all outcomes across both time periods.

Across models, the spatial-to-random variability ratio indicated that the CAR spatial random effect explained 62.2%–99.9% of the overall error variance, indicating a very high degree of spatial dependence in these models and the importance of correcting for spatial random effects. Not accounting for these effects can result in increased type I error.

## 4. Discussion

This study contributes to the paucity of literature examining the associations between housing eviction and alcohol-related outcomes (Smith et al., 2024). In particular, this paper is one of the first to examine the association between eviction filings and AUD, finding a consistently well-supported relationship between filings and increased inpatient hospitalizations for AUD across a large, geographically heterogeneous region across years. A 1% increase in eviction filings is associated with a 1.1%–1.4% increase in AUD hospitalizations, which is concerning as some ZIP codes experience considerably higher filing rates than others (e.g., in one ZIP code in 2018, 65.90% of rental households received filings, over thirteen times the statewide mean of 4.70%). Although a substantial number of studies have shown that eviction and eviction threat are associated with increased risk of suicide ideation and mortality (Elbogen et al., 2021; Guzman-Parra et al., 2023; Peek-Asa et al., 2021; Rojas and Stenberg, 2016), we did not find an association between filings and self-harm hospitalizations. This lack of association may indicate that the mechanisms linking eviction and suicide operate at the individual level more so than the ecological level or may be due to limitations of inpatient hospitalization data.

Our study also contrasts with prior literature in that we did not observe an association between filings and IPV hospitalizations nor assault hospitalizations prior to the pandemic (2018–2019); however, these associations were positive and well-supported during the pandemic (2020–2022). Researchers have previously noted that the COVID-19 pandemic shared similarities with a natural disaster in that it led to widespread social disruption and economic precarity, increasing the risk of interpersonal violence (O'Neill et al., 2023). The COVID-19 pandemic also exacerbated existing resource inequities, creating conditions in which economic and psychosocial stressors, such as eviction, could more easily lead to violence in communities (Quinn et al., 2023; Schleimer et al., 2022). During COVID-19, IPV survivors experienced decreased social support (e.g., less access to support networks because of quarantine) and reduced access to safe places and supportive services (including housing supports) (Ragavan et al., 2022). A stronger association between eviction filings and hospitalizations for assault and IPV may reflect an increase in severe cases of interpersonal violence in the context of increased psychosocial stress and decreased access to social support, resources for basic needs, and safe places. Increased social isolation in conjunction with changes in drinking patterns (e.g., more drinking at home as opposed in public spaces) could also help explain why the unemployment rate was associated with increased AUD hospitalizations during the pandemic, but not before (Castaldelli-Maia et al., 2021; Wardell et al., 2020).

The findings of this study have several implications for future research, practice, and policy. First, this study highlights the potential for preventing alcohol-related harms, especially AUD, through eviction prevention. In the U.S., an estimated 7.6 million people (nearly 7% of rental households) receive eviction filings per year, reflecting the widespread exposure of eviction threat (Graetz et al., 2023; Gromis et al., 2022). A classic public health parable tells the story of a person jumping into a river to save a person only to find that another needs help, and another after that; so consumed by saving people already in the river, the person cannot address the reason why so many people are falling into the river in the first place (McKinlay, 1979). By preventing housing insecurity and its negative health ramifications – including those related to alcohol – eviction prevention has the potential to serve as a form of “upstream” prevention. Future studies should to evaluate the impacts of eviction prevention strategies (e.g., rental assistance, civil legal aid) on alcohol and other substance use related outcomes, extending research showing that eviction moratoria are associated with improved mental health outcomes (Leifheit et al., 2021).

This research also has important health equity implications. In the U. S., housing eviction is part of a legacy of housing discrimination that perpetuates the displacement of and disinvestment in lower income, predominantly Black communities (Desmond, 2012; Fullilove, 2016; Graetz et al., 2023). Although Black individuals constitute 18.6% of all renters, they account for 51.1% of those impacted by eviction filings and 43.4% of those forced to move due to eviction (Graetz et al., 2023). The proportion of Black rental households in a neighborhood is more strongly associated with evictions than other neighborhood-level socioeconomic indicators (e.g., income level, rent burden, unemployment) (Han, 2023). Populations with lower incomes and minoritized racial and ethnic identities experience a greater burden of alcohol-related harms and problems despite having alcohol consumption levels similar or below that of higher income, White populations in the U.S. (Boyd et al., 2021; Keyes et al., 2012b; Shield et al., 2013; Zapolski et al., 2014). Structural and environmental drivers of these alcohol inequities remain underexamined (Boyd et al., 2022). Our findings that housing eviction filing rates are associated with hospitalizations for alcohol-attributable harms may indicate that housing eviction is a driver of the disproportionate impacts of alcohol borne by those with lower incomes and minoritized racial and ethnic identities. Future research should evaluate whether the impacts of eviction prevention efforts vary by racial and socioeconomic status and whether these efforts are associated with reduced disparities in alcohol-related outcomes.

From a policy perspective, this research underscores the importance of investing in upstream efforts to reduce housing insecurity in the U.S. Many housing assistance programs funded by the U.S. Department of Housing and Urban Development (HUD) prioritize chronic homelessness, defined as living in a shelter or place not fit for human habitation for at least 12 months, excluding those who may be experiencing less severe forms of instability (e.g., those who have received an eviction notice but not forced to move yet) (U.S. Department of Housing and Urban Development, 2025a). Strengthening HUD's Emergency Solutions Grants Program, which includes rental assistance and utilities assistance, and ensuring these programs are accessible by those who are at risk for receiving an eviction notice may have a sizeable impact on alcohol-related morbidity. Safeguarding federal housing protections for IPV survivors and funding for emergency IPV shelters, which has decreased in recent years, is also crucial (Office for Victims of Crime, 2025; U.S. Department of Housing and Urban Development, 2025b). On a community level, this research speaks to the public health importance of organizing and advocacy efforts to increase the power of tenants and combat community uprooting by, for example, limiting the privatization of public housing (i.e., limiting the role of corporate landlords who are more likely to evict) and increasing community land ownership (e.g., through community land trusts and cooperative housing) (McClymon et al., 2022). With greater awareness of the potential links between alcohol-attributable harms and housing eviction, public health and substance use researchers and practitioners may be better poised to support housing advocacy efforts; engagement in state- and local-level advocacy is important as eviction-related laws vary widely within the U.S. (Legal Services Corporation, 2025).

This study has several strengths and limitations. First, inpatient hospitalizations are likely to capture severe alcohol-related cases and miss those that are treated in other care settings (e.g., outpatient or emergency) or untreated (e.g., because of lack of access to health care or because they result in death before care can be accessed). This is particularly a concern for the acute harms we included as outcomes, namely assault, IPV and self-harm; this analysis only captures severe cases (i.e., those necessitating an inpatient stay) that providers have chosen to identify in medical records. Using secondary diagnosis codes (as opposed to only primary diagnosis codes) to capture hospitalizations for the outcomes of interest has the benefit of capturing more cases; however, there may be variability by condition, provider type, and patient demographic in the extent to which these codes are applied when not the primary reason for hospitalization. Second, eviction filing records likely underestimate the impacts of eviction as they do not capture informal evictions (e.g., when tenants leave after being threatened with a filing but before they receive an eviction notice). Despite these limitations, both sets of data are consistently collected across time and space at the ZIP code level, enabling spatial analysis at a finer spatial resolution than at the county or state level. Third, while these results indicate that there are positive associations between eviction filings and some types of alcohol-related hospitalizations at the ZIP code level, this does not necessarily mean that these associations exist at the individual level (e.g., that experiencing an eviction filing increases an individual's risk for being hospitalized for AUD). There is nonetheless value in understanding the potential population health impacts of reducing eviction filings at a ZIP code level. In the future, multilevel analyses could help parse the potential impacts of experiencing an eviction filing vs. living in a community with a high eviction filing rate.

This study used ecological panel data and thus did not examine longitudinal trends (e.g., changes within ZIP codes over time). Although we controlled for fixed effects, unmeasured confounding is possible and we cannot make causal conclusions. This is especially true during the pandemic (2020–2022), a period of time of rapid changes that may have affected both eviction filings and alcohol-related hospitalizations in variety of different ways. For example, increased COVID-19 cases may have been positively related to eviction risk and/or alcohol-related outcomes, while the provision of financial aid to individuals and businesses (e.g., emergency rental relief, assistance for small businesses, unemployment assistance) may have reduced both eviction risk and/or alcohol-related outcomes. It is challenging to control for these and other potentially countervailing influences given their breadth and a lack of consistently collected, geographically well-resolved data measuring their impacts. It is especially important to consider pandemic-related confounding in the context of eviction filings and hospitalizations for IPV and assault as these associations were only well-supported in 2020–2022. Additional studies are needed to examine whether these associations remain well-supported in years beyond the pandemic.

We examined eviction as an exposure and alcohol-related hospitalizations as our outcome to explore the potential for leveraging housing policy and programs for upstream public health prevention; however, the associations between eviction and alcohol-related impacts could exist in the reverse direction. For example, alcohol-related problems can include negative employment-related impacts, which could in turn generate financial strain and rental instability among larger community networks (Schwartz et al., 2024; White and Labouvie, 1987). Furthermore, assault and IPV are associated with subsequent eviction risk (Baker et al., 2003; Desmond and Gershenson, 2017). Longitudinal mediation analyses, especially those that account for time varying confounding, could be helpful for elucidating how patterns of housing insecurity relate to trajectories of alcohol use and related harms. Since alcohol use, related harms, and housing eviction may function in a cyclical, reinforcing manner, complex systems science approaches, which facilitate the examination of such relationships, could be particularly fruitful.

## Supplementary Material

Supplement

## Figures and Tables

**Table 1:** Characteristics of Pennsylvania ZIP Codes by Year in 2018-2019 vs. 2020-2022.

	2018-2019 (N = 2901 ZIP Code-Years)					2020-2022 (N = 4356 ZIP Code-Years)				
	Mean	Median	SD	Min	Max	Mean	Median	SD	Min	Max

Total population, N	8818.59	3872.94	11372.89	5.00	71271.25	8896.87	3788.08	11534.08	3.98	68805.49
Alcohol use disorder hospitalizations, N	51.96	17.00	88.21	0.00	888.00	47.67	16.00	79.56	0.00	754.00
Alcohol use disorder hospitalizations, %	0.51	0.43	0.50	0.00	15.05	0.47	0.40	0.44	0.00	11.09
Assault hospitalizations, N	0.57	0.00	2.09	0.00	37.00	0.40	0.00	1.62	0.00	30.00
Assault hospitalizations, %	0.01	0.00	0.02	0.00	0.70	0.00	0.00	0.02	0.00	0.45
Intimate partner violence hospitalizations, N	1.56	0.00	5.41	0.00	111.00	1.54	0.00	4.84	0.00	103.00
Intimate partner violence hospitalizations, %	0.02	0.00	0.05	0.00	0.88	0.02	0.00	0.04	0.00	1.00
Self-harm hospitalizations, N	0.51	0.00	1.68	0.00	27.00	0.51	0.00	1.73	0.00	26.00
Self-harm hospitalizations, %	0.01	0.00	0.02	0.00	0.35	0.01	0.00	0.03	0.00	1.00
Eviction filing rate, % of rental households	4.70	3.34	5.24	0.00	65.91	3.33	2.25	4.09	0.00	58.73
Male, %	49.85	49.69	2.58	40.37	82.25	50.11	49.94	2.57	38.53	77.91
Age										
Under 18, %	20.32	20.25	3.52	1.20	32.81	20.10	20.14	3.77	0.57	35.23
18-24, %	8.35	7.43	5.29	2.63	87.67	8.19	7.31	5.19	2.56	89.53
25-44, %	22.93	22.22	4.19	5.42	62.04	22.99	22.16	4.53	4.31	64.99
45-64, %	29.15	29.56	3.55	2.64	60.84	28.43	28.82	3.61	3.17	42.26
65 and over, %	19.24	19.50	3.67	1.66	40.95	20.28	20.58	4.02	1.51	37.94
Race/Ethnicity										
Hispanic or Latino, %	3.88	1.89	6.48	0.00	68.70	4.33	2.15	6.84	0.00	69.47
Non-Hispanic or Latino Black, %	4.93	1.21	10.94	0.00	88.60	4.78	1.22	10.64	0.00	89.07
Non-Hispanic or Latino White, %	87.81	93.92	15.80	4.19	100.00	86.56	92.47	15.84	4.53	99.87
All Other Races & Ethnicities, %	3.38	2.19	3.29	0.00	24.95	4.33	3.13	3.64	0.00	27.23
Married, %	25.58	27.03	5.02	0.54	36.38	25.48	26.64	5.00	0.37	37.16
Population with college degree, %	15.44	13.28	7.21	4.67	61.17	16.23	13.98	7.25	4.92	43.43
Unemployed, %	3.14	2.91	1.21	0.52	10.84	3.06	2.84	1.27	0.37	11.24
Income below poverty level, %	11.15	10.28	5.93	1.54	50.23	10.51	9.65	5.67	1.02	50.78
Median income, per $10k	6.25	5.73	1.99	1.81	19.74	7.05	6.42	2.33	2.03	22.95
Rent burden, % of rental households	36.53	36.12	9.74	0.87	79.04	35.34	34.70	10.26	2.03	87.61
Vacant housing, % of housing units	20.51	17.69	11.00	3.12	81.98	20.21	17.15	11.37	2.43	79.57
Insured, %	92.00	93.64	5.87	34.08	99.23	92.32	94.11	5.90	37.78	99.62
All hospitalizations, per 100 people	2.92	2.90	1.27	0.00	14.26	2.60	2.56	1.40	0.00	31.69
Presence of a hospital	380	13.10				552	12.67			
Presence of a substance use and mental health treatment facility	726	25.03				1084	24.89			
Alcohol Outlet density, per mi^2^	2.37	0.13	20.42	0.00	910.43	1.97	0.13	11.25	0.00	248.09
Proportion of off-premise alcohol outlets	0.12	0.00	0.18	0.00	1.00	0.12	0.00	0.18	0.00	1.00

SD = standard deviation.

All Other Races & Ethnicities includes those identifying as Non-Hispanic or Latino American Indian and Alaska Native, Asian, Native Hawaiian and Other Pacific Islander, and multiple races; statistics associated with presence of a hospital and presence of a substance use and mental health treatment facility are the number and percent of ZIP codes with at least one hospital or SUMH treatment facility, respectively.

**Table 2:** Relative rates and 95% credible intervals of hospitalization for AUD and assault estimated from spatial misalignment models for 2018-2019 and 2020-2022.

	AUD RR (95% CI)		Assault RR (95% CI)	
	2018-2019	2020-2022	2018-2019	2020-2022

Eviction filing rate, % per rental households	1.014 (1.011, 1.018)^a^	1.011 (1.007, 1.014)^a^	1.015 (0.993, 1.038)	1.033 (1.002, 1.065)^a^
Year (ref: 2018 for 2018-2019; 2020 for 2020-2022)				
2019	1.021 (0.996, 1.047)		1.166 (0.907, 1.501)	
2021		1.057 (1.031, 1.082)^a^		1.140 (0.864, 1.504)
2022		1.069 (1.038, 1.101)^a^		0.723 (0.515, 1.014)
Population density, people per sq mile/100 (ref: Quintile 1)				
Quintile 2	1.007 (0.951, 1.066)	0.974 (0.928, 1.023)	0.654 (0.437, 0.98)^a^	0.815 (0.573, 1.161)
Quintile 3	1.047 (0.981, 1.119)	1.060 (1.001, 1.122)^a^	0.7 (0.454, 1.081)	0.879 (0.596, 1.297)
Quintile 4	1.022 (0.947, 1.104)	1.018 (0.953, 1.088)	0.516 (0.314, 0.85)^a^	0.910 (0.589, 1.405)
Quintile 5	1.001 (0.914, 1.097)	0.984 (0.909, 1.065)	0.5 (0.282, 0.887)^a^	1.048 (0.639, 1.721)
Race/Ethnicity, % (ref: Non-Hispanic or Latino White)				
Hispanic or Latino	0.998 (0.995, 1.001)	0.996 (0.993, 0.999)^a^	0.973 (0.957, 0.991)^a^	0.973 (0.956, 0.992)^a^
Non-Hispanic or Latino Black	0.998 (0.996, 1.000)	0.999 (0.997, 1.001)	0.997 (0.987, 1.007)	1.002 (0.991, 1.014)
All Other Races & Ethnicities	0.997 (0.991, 1.003)	0.994 (0.989, 0.999)^a^	0.996 (0.966, 1.027)	1.001 (0.969, 1.036)
Age, % (ref: 45-64)				
Under 18, %	0.976 (0.968, 0.985)^a^	0.982 (0.975, 0.989)^a^	1.012 (0.960, 1.068)	1.020 (0.973, 1.069)
18-24, %	0.964 (0.956, 0.97)^a^	0.968 (0.963, 0.973)^a^	0.959 (0.921, 0.997)^a^	0.973 (0.941, 1.007)
24-44 %	0.981 (0.972, 0.99)^a^	0.989 (0.982, 0.996)^a^	1.017 (0.972, 1.063)	1.011 (0.969, 1.055)
65+, %	0.973 (0.964, 0.983)^a^	0.981 (0.974, 0.989)^a^	1.012 (0.956, 1.071)	0.997 (0.947, 1.049)
Married, %	0.956 (0.946, 0.967)^a^	0.965 (0.957, 0.972)^a^	0.923 (0.869, 0.981)^a^	0.933 (0.885, 0.984)^a^
Unemployed, %	0.976 (0.958, 0.995)^a^	1.021 (1.007, 1.036)^a^	1.031 (0.931, 1.142)	1.046 (0.958, 1.141)
Income below poverty level, %	1.006 (1.000, 1.012)	1.007 (1.002, 1.012)^a^	1.023 (0.994, 1.052)	1.010 (0.982, 1.039)
Median income, per $10k	0.977 (0.962, 0.994)^a^	0.975 (0.963, 0.988)^a^	1.004 (0.910, 1.108)	0.910 (0.829, 1.000)
Rent burden, % of rental households	0.998 (0.995, 1.000)	0.999 (0.997, 1.000)	1.001 (0.987, 1.016)	0.997 (0.984, 1.009)
Vacant housing, % of housing units	1.003 (1,000, 1.006)	1.002 (0.999, 1.005)	1.010 (0.993, 1.028)	0.994 (0.977, 1.011)
Insured, %	1.018 (1.014, 1.021)^a^	1.014 (1.011, 1.017)^a^	1.007 (0.984, 1.03)	0.991 (0.969, 1.013)
All hospitalizations, per 100 people	1.289 (1.267, 1.311)^a^	1.281 (1.265, 1.300)^a^	1.293 (1.161, 1.441)^a^	1.157 (1.051, 1.274)^a^
Hospital in ZIP code	1.029 (0.995, 1.064)	1.054 (1.025, 1.084)^a^	0.974 (0.827, 1.149)	1.326 (1.117, 1.575)^a^
Substance Use and Mental Health Treatment Facility in ZIP code	1.030 (0.999, 1.062)	1.046 (1.019, 1.073)^a^	1.077 (0.915, 1.266)	1.035 (0.880, 1.218)
Outlet density, per sq2	1.002 (1.001, 1.003)^a^	1.004 (1.003, 1.005)^a^	1.005 (1.003, 1.008)^a^	1.007 (0.998, 1.015)
Proportion of outlets off premise	1.184 (1.094, 1.281)^a^	1.115 (1.043, 1.192)^a^	1.433 (0.798, 2.575)	0.907 (0.504, 1.632)
**Random effects**				
Spatial random effect (SD CAR process)	0.245 (0.251, 0.257)	0.339 (0.345, 0.352)	1.44 (1.464, 1.489)	1.741 (1.765, 1.790)
ZIP code-level random effects (SD)	0.146 (0.169, 0.195)	0.0390 (0.076, 0.12)	0.014 (0.035, 0.108)	0.014 (0.035, 0.102)
Spatial-to-total random variability ratio	0.622 (0.687, 0.747)	0.892 (0.954, 0.987)	0.995 (0.999, 1.000)	0.997 (0.999, 1.000)

AUD = Alcohol Use Disorder; IPV = Intimate Partner Violence; RR= Relative Rate; CI = Credible Interval; CAR = Conditional Autoregressive; SD = Standard Deviation.

All Other Races & Ethnicities includes those identifying as Non-Hispanic or Latino American Indian and Alaska Native, Asian, Native Hawaiian and Other Pacific Islander, and multiple races.

a Finding is well supported by the data as indicated by the credible interval excluding 1.

**Table 3:** Relative rates and 95% credible intervals of hospitalization for IPV and self-harm estimated from spatial misalignment models for 2018-2019 and 2020-2022.

	IPV RR (95% CI)		Self-Harm RR (95% CI)	
	2018-2019	2020-2022	2018-2019	2020-2022

Eviction filing rate, % per rental households	0.995 (0.981, 1.010)	1.015 (1.002, 1.028)^a^	0.994 (0.969, 1.020)	1.007 (0.977, 1.037)
Year (ref: 2018 for 2018-2019; 2020 for 2020-2022)				
2019	1.059 (0.959, 1.170)		0.712 (0.562, 0.900)^a^	
2021		1.106 (0.999, 1.224)		1.446 (1.121, 1.865)^a^
2022		1.252 (1.115, 1.406)^a^		0.861 (0.638, 1.161)
Population density, people per sq mile/100 (ref: Quintile 1)				
Quintile 2	0.868 (0.710, 1.061)	1.070 (0.902, 1.271)	0.588 (0.411, 0.841)^a^	1.065 (0.788, 1.438)
Quintile 3	0.770 (0.607, 0.978)^a^	1.103 (0.906, 1.344)	0.676 (0.451, 1.012)	1.100 (0.783, 1.543)
Quintile 4	0.636 (0.479, 0.846)^a^	0.929 (0.739, 1.168)	0.538 (0.331, 0.876)^a^	0.890 (0.602, 1.317)
Quintile 5	0.553 (0.390, 0.784)^a^	0.762 (0.579, 1.002)	0.446 (0.250, 0.795)^a^	0.724 (0.458, 1.145)
Race/Ethnicity, % (ref: Non-Hispanic or Latino White)				
Hispanic or Latino	0.988 (0.978, 0.999)^a^	0.991 (0.984, 0.999)^a^	0.973 (0.952, 0.995)^a^	0.970 (0.950, 0.992)^a^
Non-Hispanic or Latino Black	0.991 (0.984, 0.998)^a^	0.994 (0.989, 1.000)	0.991 (0.977, 1.004)	0.993 (0.981, 1.005)
All Other Races & Ethnicities	0.997 (0.975, 1.019)	0.996 (0.980, 1.012)	0.997 (0.959, 1.038)	1.029 (0.996, 1.064)
Age, % (ref: 45-64)				
Under 18, %	0.988 (0.953, 1.023)	1.000 (0.976, 1.024)	1.010 (0.951, 1.071)	1.005 (0.962, 1.05)
18-24, %	0.962 (0.936, 0.988)^a^	0.982 (0.964, 1.000)	0.956 (0.916, 0.998)^a^	0.955 (0.925, 0.987)^a^
24-44 %	0.972 (0.942, 1.004)	1.011 (0.989, 1.034)	0.992 (0.943, 1.045)	0.983 (0.944, 1.024)
65+, %	0.952 (0.918, 0.989)^a^	0.979 (0.954, 1.005)	0.962 (0.903, 1.024)	0.991 (0.946, 1.038)
Married, %	0.952 (0.916, 0.990)^a^	0.970 (0.946, 0.997)^a^	0.953 (0.893, 1.017)	0.958 (0.913, 1.005)
Unemployed, %	1.033 (0.965, 1.106)	1.047 (1.002, 1.094)^a^	0.954 (0.845, 1.078)	1.020 (0.937, 1.111)
Income below poverty level, %	1.018 (0.998, 1.039)	1.020 (1.004, 1.037)^a^	1.026 (0.993, 1.061)	1.011 (0.983, 1.04)
Median income, per $10k	0.980 (0.920, 1.045)	0.947 (0.908, 0.990)^a^	1.042 (0.932, 1.164)	1.052 (0.970, 1.141)
Rent burden, % of rental households	1.000 (0.992, 1.008)	1.005 (0.999, 1.011)	0.997 (0.983, 1.012)	1.008 (0.997, 1.019)
Vacant housing, % of housing units	1.001 (0.989, 1.013)	0.992 (0.983, 1.001)	1.007 (0.987, 1.026)	1.015 (0.998, 1.033)
Insured, %	1.018 (1.003, 1.033)^a^	1.012 (1.001, 1.022)^a^	0.997 (0.974, 1.020)	0.995 (0.974, 1.016)
All hospitalizations, per 100 people	1.246 (1.160, 1.339)^a^	1.240 (1.182, 1.302)^a^	1.265 (1.123, 1.423)^a^	1.231 (1.132, 1.339)^a^
Hospital in ZIP code	1.131 (1.012, 1.264)^a^	1.073 (0.980, 1.172)	1.099 (0.912, 1.323)	1.126 (0.963, 1.318)
Substance Use and Mental Health Treatment Facility in ZIP code	1.101 (0.987, 1.229)	0.969 (0.888, 1.059)	1.223 (1.024, 1.458)^a^	0.991 (0.857, 1.147)
Outlet density, per mi2	1.002 (0.999, 1.006)	1.005 (1.002, 1.008)^a^	1.004 (1.000, 1.008)	1.002 (0.992, 1.012)
Proportion of outlets off premise	1.202 (0.881, 1.639)	1.045 (0.812, 1.346)	1.655 (0.950, 2.881)	0.940 (0.575, 1.537)
**Random effects**				
Spatial random effect (SD CAR process)	0.850 (0.870, 0.89)	0.828 (0.844, 0.860)	1.585 (1.615, 1.645)	1.849 (1.874, 1.899)
ZIP code-level random effects (SD)	0.015 (0.033, 0.085)	0.014 (0.032, 0.084)	0.014 (0.035, 0.108)	0.016 (0.034, 0.085)
Spatial-to-total random variability ratio	0.991 (0.999, 1.000)	0.990 (0.999, 1.000)	0.996 (0.999, 1.000)	0.998 (0.999, 1.000)

AUD = Alcohol Use Disorder; IPV = Intimate Partner Violence; RR= Relative Rate; CI = Credible Interval; CAR = Conditional Autoregressive; SD = Standard Deviation.

All Other Races & Ethnicities includes those identifying as Non-Hispanic or Latino American Indian and Alaska Native, Asian, Native Hawaiian and Other Pacific Islander, and multiple races.

a Finding is well supported by the data as indicated by the credible interval excluding 1.

## Data Availability

The authors do not have permission to share data.

## Footnotes

1 Philadelphia eviction court filings are not recorded differently from elsewhere in the state, but just housed separately since Philadelphia has its own

2 SUMH facility listings were downloaded at the end of 2024 and assumed to be stable over time. Hospital locations were available for September 2018, December 2019, December 2020, and March 2022 and thus year-specific estimates were used for those years. Hospital locations for 2021 were not available, so December 2020 estimates were used for this year.
