# Supplementary material for: A spatial analysis of the associations between housing eviction and alcohol-related hospitalizations in Pennsylvania
Source: Health Place. Author manuscript; Available in PMC 2026 Jun 26. (PMC13306642; doi:10.1016/j.healthplace.2026.103632)
Supplement: Supplement [file NIHMS2178811-supplement-Supplement.docx]

**Supplement. ICD-10 Diagnosis Codes Used to Identify Alcohol-Related Hospitalizations**

| **Hospitalization Type** | **ICD-10 Codes** |
| --- | --- |
| Alcohol use disorder^1^ | F10* |
| Assault and homicide^2^ | X92-Y08, Y09 |
| Intimate partner violence^3^ | T74.11* , T74.21* , T74.31*, Y07.0*, Z69.11*, Z63.0* |
| Suicide/self-inflicted injury^2^ | X71-X83 |

^1.^ The Centers for Disease Control (CDC) Alcohol-Related Disease Impact (ARDI) System (<https://www.cdc.gov/alcohol/ardi/alcohol-related-icd-codes.html>) defines alcohol abuse and dependence as ICD-10 codes F10.0-F10.2. In our inpatient hospital discharge data, hospitalization with F10.0-F10.2 constituted 98.3% of all hospitalizations with any F10* code (based on primary and secondary diagnoses). Other researchers (Bernstein et al., 2023; Manca & Lewsey, 2024; Riedel et al., 2024; Suen et al., 2022) have commonly use F10* (i.e., including F10.3-F10.9), including alcoholic psychosis, in their definitions of AUD, thus we also included these codes in our definition as well.

^2.^ Codes based on Centers for Disease Control (CDC) Alcohol-Related Disease Impact (ARDI) System (<https://www.cdc.gov/alcohol/ardi/alcohol-related-icd-codes.html>)

^3.^ Codes based on 2024 Uniform Data Reporting Standards, World Health Organization ICD-10 definitions, and peer-reviewed literature (Auger et al., 2021; Health Resources and Services Administration, 2024; Radhakrishnan et al., 2024; Rebbe et al., 2023; World Health Organization, 2019).

**References**

Auger,  Nathalie, Low,  Nancy, Lee,  Ga Eun, Ayoub,  Aimina, & Luu,  Thuy Mai. (2021). Pregnancy Outcomes of Women Hospitalized for Physical Assault, Sexual Assault, and Intimate Partner Violence. *Journal of Interpersonal Violence*, *37*(13–14), NP11135–NP11135. https://doi.org/10.1177/0886260520985496

Bernstein, E. Y., Baggett, T. P., Trivedi, S., Herzig, S. J., & Anderson, T. S. (2023). Pharmacologic Treatment Initiation Among Medicare Beneficiaries Hospitalized With Alcohol Use Disorder. *Annals of Internal Medicine*, *176*(8), 1137–1139. https://doi.org/10.7326/M23-0641

Health Resources and Services Administration. (2024). *Uniform Data System Health Center Data Reporting Requirements*. https://bphc.hrsa.gov/sites/default/files/bphc/data-reporting/2024-uds-manual.pdf

Manca, F., & Lewsey, J. (2024). Previous psychiatric hospitalizations as risk factors for single and multiple future alcohol-related hospitalizations in patients with alcohol use disorders. *Addiction*, *119*(2), 291–300. https://doi.org/https://doi.org/10.1111/add.16352

Radhakrishnan, P., Bekele, F., & Raju, A. (2024). *Potential Implications Of Federal Regulations For Survivors Of Intimate Partner Violence*. https://www.healthaffairs.org/content/forefront/potential-implications-federal-regulations-survivors-intimate-partner-violence

Rebbe, R., Adhia, A., Eastman, A. L., Chen, M., & Winn, J. (2023). The Measurement of Intimate Partner Violence Using International Classification of Diseases Diagnostic Codes: A Systematic Review. *Trauma, Violence & Abuse*, *24*(4), 2165–2180. https://doi.org/10.1177/15248380221090977

Riedel, O., Braitmaier, M., Dankhoff, M., Haug, U., Klein, M., Zachariassen, W., & Hoyer, J. (2024). Alcohol use disorders after bariatric surgery: a study using linked health claims and survey data. *International Journal of Obesity*, *48*(11), 1656–1663. https://doi.org/10.1038/s41366-024-01606-3

Suen, L. W., Makam, A. N., Snyder, H. R., Repplinger, D., Kushel, M. B., Martin, M., & Nguyen, O. K. (2022). National Prevalence of Alcohol and Other Substance Use Disorders Among Emergency Department Visits and Hospitalizations: NHAMCS 2014-2018. *Journal of General Internal Medicine*, *37*(10), 2420–2428. https://doi.org/10.1007/s11606-021-07069-w

World Health Organization. (2019). *ICD-10 Version:2019*. https://icd.who.int/browse10/2019/en
